# Retraction: Kim et al. Advantage of Species Diversification to Facilitate Sustainable Development of Aquaculture Sector. *Biology* 2022, *11*, 368

**DOI:** 10.3390/biology11040509

**Published:** 2022-03-25

**Authors:** Dae-Young Kim, Surendra Krushna Shinde, Avinash Ashok Kadam, Rijuta Ganesh Saratale, Ganesh Dattatraya Saratale, Manu Kumar, Asad Syed, Ali H. Bahkali, Gajanan Sampatrao Ghodake

**Affiliations:** 1 Department of Biological and Environmental Science, College of Life Science and Biotechnology, Dongguk University-Seoul, 32 Dongguk-ro, Ilsandong-gu, Goyang-si 10326, Gyeonggi-do, Korea; sbpkim@dongguk.edu (D.-Y.K.); shindesurendra9@gmail.com (S.K.S.); 2 Research Institute of Biotechnology and Medical Converged Science, Dongguk University-Seoul, 32 Dongguk-ro, Ilsandong-gu, Goyang-si 10326, Gyeonggi-do, Korea; avikadam2010@gmail.com (A.A.K.); rijutaganesh@gmail.com (R.G.S.); 3 Department of Food Science and Biotechnology, College of Life Science and Biotechnology, Dongguk University-Seoul, 32 Dongguk-ro, Ilsandong-gu, Goyang-si 10326, Gyeonggi-do, Korea; gdsaratale@gmail.com; 4 Department of Life Science, College of Life Science and Biotechnology, Dongguk University-Seoul, 32 Dongguk-ro, Ilsandong-gu, Goyang-si 10326, Gyeonggi-do, Korea; manukumar007@gmail.com; 5 Department of Botany and Microbiology, College of Science, King Saud University, P.O. Box 2455, Riyadh 11451, Saudi Arabia; assyed@ksu.edu.sa (A.S.); abahkali@ksu.edu.sa (A.H.B.)

*Biology* retracts the article “Advantage of Species Diversification to Facilitate Sustainable Development of Aquaculture Sector” cited above [1]. 

Following publication, a reader raised concerns to the editorial office regarding the overlap of ideas and research presented in a previously published article [2], including the title, concepts, figures, and glossary. The authors agreed and requested retraction. 

Adhering to our complaints procedure, an investigation was conducted by the Editorial Board and the Academic Editor (Prof. Dr. Ewald Schnug) in collaboration with the editorial office, who confirmed the extent of the overlap. The authors would like to apologize for any inconvenience caused to the readers.

This retraction was approved by the Editor-in-Chief of the journal *Biology*. 

We apologize to the readers of *Biology*.
